# Reassessing the Application of the Haller Index as a Surgical Indicator for Pectus Excavatum

**DOI:** 10.7759/cureus.105884

**Published:** 2026-03-26

**Authors:** Bryan J Calderin Barreto, Glorimar Salcedo Martir, Maria L Nieves Tirado, Ivan R Iriarte, Norman Ramírez-Lluch, Victor N Ortiz Justiniano

**Affiliations:** 1 Pediatrics, Puerto Rico Children's Hospital, Bayamon, PRI; 2 Family Medicine, Mayaguez Medical Center, Mayaguez, PRI; 3 Pediatric Orthopedic Surgery, Mayaguez Medical Center, Mayaguez, PRI; 4 Pediatric Surgery, Auxilio Mutuo Pediatrico, San Juan, PRI

**Keywords:** chest wall deformity, haller index, hispanic population, pectus excavatum, pulmonary function tests, surgical candidacy

## Abstract

Pectus excavatum, or “funnel chest,” is the most common congenital chest wall deformity, characterized by posterior depression of the sternum and adjacent costal cartilages. Although frequently identified in childhood, the deformity often progresses during adolescence and may lead to exercise intolerance, respiratory symptoms, and psychosocial distress. Traditionally, surgical indications have been guided by the Haller index, a radiographic metric derived from chest computed tomography (CT) scans, although its reliability in predicting functional compromise remains debated. This retrospective study evaluated 41 Hispanic pediatric patients with pectus excavatum (mean age 14.1 years), of whom 36 of 41 (88%) were male, who were seen at Puerto Rico Children’s Hospital between 2015 and 2021. Spirometry values, including forced vital capacity (FVC) and forced expiratory volume in one second (FEV₁), were compared across Haller index categories (<3.25 vs ≥ 3.25). No statistically significant correlation was found between the Haller index and individual spirometry parameters, consistent with weak Pearson correlation coefficients (r = -0.26 for FVC and r = -0.14 for FEV₁). Receiver operating characteristic (ROC) analysis demonstrated limited ability of the Haller index to identify reduced FVC (AUC = 0.63). These findings suggest that the Haller index alone is insufficient as a standalone surgical indicator for pectus excavatum. A comprehensive evaluation incorporating dynamic cardiopulmonary assessment, psychological considerations, and refined imaging tools such as the correction index may better inform surgical decision-making.

## Introduction

Pectus excavatum, commonly referred to as “funnel chest,” is the most prevalent congenital chest wall deformity, presenting as a posterior depression of the sternum and adjacent costal cartilages [1]. This deformity occurs in approximately one to eight per 1,000 live births [2,3]. It demonstrates a 4:1 male-to-female preponderance and may be familial in up to 27% of cases. Although usually isolated, pectus excavatum can be associated with syndromic and musculoskeletal disorders, including Marfan syndrome, Noonan syndrome, Ehlers-Danlos syndrome, and Prune-belly syndrome [3].

Patients with pectus excavatum may present with mild to moderate exercise intolerance, chest pain with exertion, recurrent respiratory infections, palpitations, and shortness of breath [4-6]. Bullying and psychological challenges are commonly reported during adolescence and early adulthood secondary to cosmetic dissatisfaction [4]. Although often identified in early childhood, the deformity typically worsens during periods of rapid growth, particularly during puberty. Histologic and structural studies suggest that abnormal costal cartilage growth and altered connective tissue architecture contribute to the pathogenesis of the deformity [7].

Computed tomography (CT) scans are commonly used to evaluate chest wall anatomy and quantify the severity of pectus excavatum deformity in children [2]. In addition, echocardiography and pulmonary function tests (PFTs) are frequently performed to assess clinical severity and detect associated conditions, including mitral valve prolapse and cardiopulmonary compromise [6,8]. Surgical correction options include minimally invasive and open techniques, such as the Nuss procedure and modified Ravitch repair [9,10], with reported favorable outcomes across pediatric and adult populations [6]. Contemporary management strategies emphasize individualized decision-making based on anatomical severity, symptom burden, and functional assessment [9].

In 1987, Haller et al. introduced a radiographic measurement, the Haller index, calculated as the ratio of the transverse chest diameter to the anteroposterior diameter at the narrowest point on CT [1]. A Haller index greater than 3.25 has traditionally been used to define severe deformity and guide surgical consideration [1]. However, this threshold was not originally derived from physiological outcome data and remains debated in contemporary management discussions [9]. Therefore, reliance on the Haller index as the sole criterion for surgical candidacy has been questioned because its correlations with pulmonary function vary across populations.

Given these considerations, this study aims to reassess the validity of the Haller index as a standalone surgical indicator for pectus excavatum. Specifically, we sought to evaluate the relationship between the Haller index and spirometric parameters in a predominantly Hispanic pediatric population, thereby informing more refined clinical decision-making.

This article was previously presented as a poster at the European Academy of Pediatrics (EAP) Congress on October 17, 2025.

## Materials and methods

This retrospective study included Hispanic pediatric patients diagnosed with pectus excavatum who were evaluated at Puerto Rico Children’s Hospital between 2015 and 2021. Demographic data, associated syndromes, radiographic measurements, and preoperative PFT results were obtained from electronic medical records. The Haller index was calculated from CT imaging as the ratio of the transverse diameter of the chest to the anteroposterior diameter at the level of greatest sternal depression.

Inclusion criteria required the availability of both CT imaging for Haller index calculation and preoperative spirometry data. Patients with prior chest wall surgery were excluded to avoid confounding postoperative changes in pulmonary function. Syndromic cases, including Marfan syndrome and Ehlers-Danlos syndrome, were included as they represent clinically relevant populations encountered in practice.

Spirometry was performed, and predicted values were calculated using Knudson's reference equations [11]. Pulmonary function results were expressed as percentages of predicted values. Abnormal pulmonary function was defined as a forced vital capacity (FVC) or forced expiratory volume in one second (FEV₁) below 80% of the predicted value.

Descriptive statistics were used to summarize patient demographics, associated syndromes, and PFT results. The Haller index was analyzed both as a continuous and a dichotomous variable, using a threshold of 3.25 (Haller < 3.25 vs. ≥ 3.25) to define elevated deformity severity. Continuous variables were expressed as mean ± standard deviation (SD). Comparisons of preoperative PFT values (FVC and FEV₁) between Haller index groups were performed using the Mann-Whitney U test, given the small sample size and distribution characteristics of the variables. Pearson correlation analysis was used to assess linear associations between the continuous Haller index and pulmonary function parameters. Receiver operating characteristic (ROC) curve analysis was conducted to evaluate the discriminative ability of the Haller index in identifying patients with abnormal pulmonary function. Statistical significance was defined as a two-tailed p-value < 0.05. Statistical analyses were performed using IBM SPSS Statistics for Windows, version 29.0 (IBM Corp., Armonk, NY). Missing data were excluded on a per-variable basis to maximize the use of available observations. Findings were interpreted in light of the limited statistical power inherent to the small cohort.

## Results

This retrospective, observational study included 41 pediatric patients with pectus excavatum (36 of 41 (88%) male; mean age, 14.1 years; SD, 3.5) who were evaluated at Puerto Rico Children’s Hospital between 2015 and 2021. All participants were Hispanic, and common comorbidities included Marfan syndrome (12%), scoliosis (12%), Ehlers-Danlos syndrome (5%), and Poland syndrome (2.4%). The mean Haller index was 3.8 (SD, 0.9), with a range of 2.5-7.2. PFTs, summarized in Table 1, demonstrated a mean FVC of 80.1% predicted and a mean FEV in one second (FEV₁) of 79.1% predicted.

**Table 1:** Preoperative pulmonary function test (PFT) results (N = 41) FVC: Forced vital capacity; FEV_1_: Forced expiratory volume in one second.

Parameters	Mean ± SD	Median	Range
FVC (% predicted)	80.1 ± 13.3	82.0	48.0-112.0
FEV₁ (% predicted)	79.1 ± 13.2	81.0	41.0-110.0

Patients were stratified by Haller index severity (Haller <3.25 vs ≥3.25). As shown in Table 2, the ≥3.25 group had lower mean spirometry values; however, these differences were not statistically significant (mean FVC: 77.6% vs 84.9%, p = 0.141; mean FEV₁: 77.7% vs 81.8%, p = 0.322).

**Table 2:** Comparison of pulmonary function tests by Haller index group (N = 41; Haller < 3.25, n = 14; Haller ≥ 3.25, n = 27) Values are presented as mean ± SD. Group comparisons were performed using the Mann-Whitney U test. FVC: Forced vital capacity; FEV_1_: Forced expiratory volume in one second.

Parameters	Haller < 3.25 (n = 14), Mean ± SD	Haller ≥ 3.25 (n = 27), Mean ± SD	U	p-value
FVC (% predicted)	84.9 ± 14.0	77.6 ± 12.4	135.5	0.141
FEV₁ (% predicted)	81.8 ± 13.8	77.7 ± 14.5	153.0	0.322

**Figure 1:**
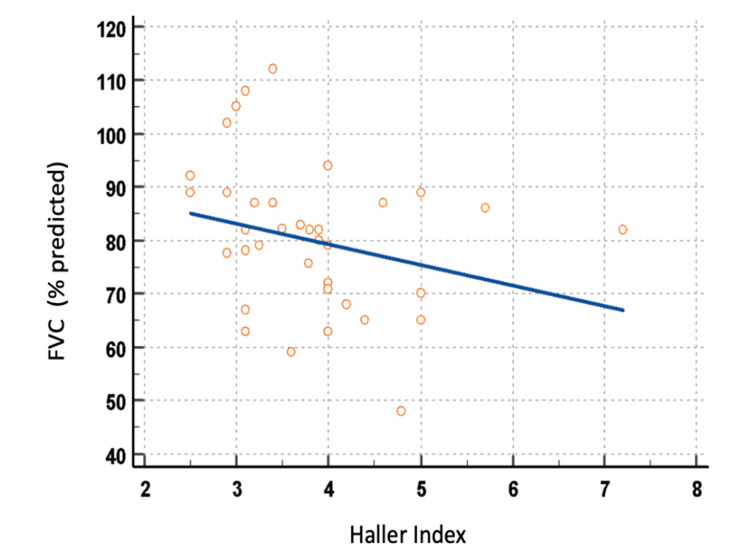
Association between Haller index and FVC (% predicted) Pearson correlation analysis demonstrated a weak negative association (r = −0.26; 95% CI: −0.53 to 0.05). FVC: Forced vital capacity.

**Figure 2:**
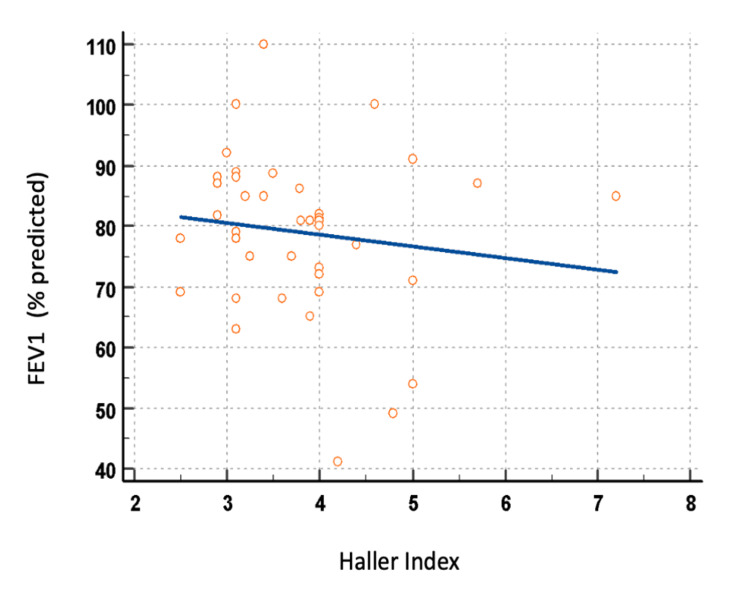
Association between Haller index and FEV₁ (% predicted) Pearson correlation analysis demonstrated a weak negative association (r = −0.14; 95% CI: −0.43 to 0.18). FEV_1_: Forced expiratory volume in one second.

To further explore the relationship between deformity severity and pulmonary function, correlation analyses were performed, treating the Haller index as a continuous variable. A weak inverse correlation was observed between the Haller index and FVC (r = −0.26, 95% CI: −0.53 to 0.05, p = 0.10; Figure 1), and a negligible correlation was observed between the Haller index and FEV₁ (r = −0.14, 95% CI: −0.43 to 0.18, p = 0.36; Figure 2).

**Figure 3:**
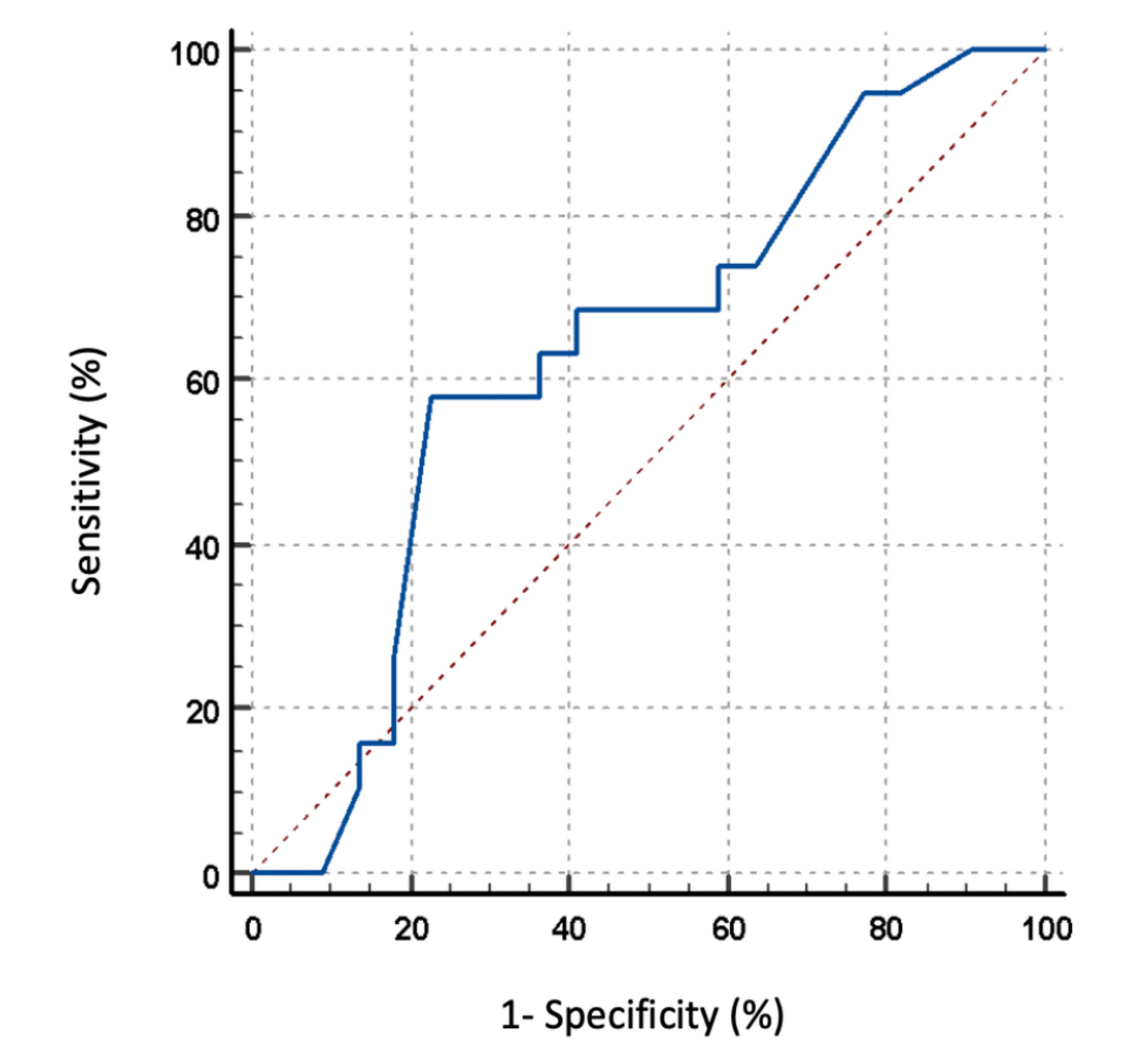
Receiver operating characteristic (ROC) curve for Haller index ROC curve evaluating the ability of the Haller index to identify abnormal pulmonary function (FVC < 80% predicted); area under the curve (AUC) = 0.63.

ROC analysis demonstrated limited discriminative ability of the Haller index for identifying reduced FVC (AUC = 0.63), as shown in Figure 3. An exploratory threshold of Haller index > 3.89 yielded higher specificity for reduced FVC; however, overall diagnostic performance remained limited. These findings suggest that even at higher Haller index values, the ability to predict reduced FVC using resting spirometry remains weak.

## Discussion

Our data demonstrate a poor relationship between the Haller index and pulmonary function in the Hispanic population studied. Before the study by Haller et al., operative repair decisions were traditionally based on subjective assessments, including abnormal chest wall dynamics, general distortion of the chest wall configuration, and caliper measurements of anterior-posterior chest diameters. In 1987, Haller et al. proposed an objective measurement, the Haller index, suggesting that a value greater than 3.25 indicated the need for surgical repair [1]. However, the 3.25 threshold was not originally derived from physiological outcome data [1] and remains debated in contemporary management discussions [9].

Several studies have reported that higher Haller index values are associated with measurable decrements in pulmonary function. For example, Zens et al. [12] found that increasing deformity severity is associated with impaired cardiopulmonary function. Similarly, Ramadan et al. [13] observed functional impairment in adolescent patients. Lawson et al. [14] demonstrated significant changes in pulmonary function before and after the Nuss procedure. Borowitz et al. [5] also reported improvements in pulmonary function and exercise response following surgical repair. Malek et al. [15] confirmed modest improvement in their meta-analysis.

Over the past decade, multiple studies have investigated the relationship between the Haller index and preoperative PFT results, yielding mixed findings [9,15]. Other investigations have found that although pulmonary dysfunction may be present, cardiac dysfunction does not consistently correlate with deformity severity. Töpper et al. [16] used cardiac magnetic resonance imaging and demonstrated limited right ventricular functional compromise in some cases, underscoring the need for comprehensive cardiopulmonary assessment rather than reliance on a single anatomical measurement.

On the other hand, the correction index has been proposed as a more anatomically precise alternative to the Haller index. By accounting for both sternal depth and the distribution of the deformity across the chest wall, the correction index may capture subtle variations in chest wall geometry that the Haller index overlooks. Poston et al. [17] suggested that the correction index may better guide surgical decision-making and establish a more physiologically meaningful threshold for operative repair. Broader global outcome analyses have further emphasized variability in long-term results after repair [18].

In our study, no statistically significant correlation was found between the Haller index and individual spirometry parameters such as FVC and FEV₁, consistent with weak Pearson correlation coefficients (r = −0.26 and r = −0.14, respectively). This suggests that spirometry values may remain preserved even in patients with more severe chest wall deformities (Haller index ≥ 3.25), underscoring the variability in physiological impact. Our findings support the need for a more comprehensive approach to evaluating surgical candidates for pectus excavatum. Although a higher Haller index was associated with a lower mean FVC in subgroup analysis, this association did not correspond to a clear linear correlation across the cohort. Thus, the Haller index may reflect anatomical severity but does not consistently predict pulmonary impairment. Several factors may contribute to this dissociation. Patients may develop compensatory respiratory mechanics that preserve spirometric values despite structural deformity. Additionally, variability in chest wall compliance and differences in intrathoracic dynamics may influence functional impact. Cardiac compression, particularly involving the right ventricle, may also contribute to symptoms that are not captured by resting spirometry. These considerations further support the need for dynamic cardiopulmonary assessment, including exercise testing and echocardiographic evaluation, when determining clinical severity and surgical candidacy. This further highlights the limitation of static imaging indices in predicting functional compromise.

This study has several limitations. First, the retrospective, single-center design limits generalizability. Additionally, potential confounding variables, such as body mass index (BMI), symptom severity, and baseline functional status, were not controlled for in this analysis, which may independently influence pulmonary function. The sample size was modest, and all patients were evaluated at a single tertiary care institution serving a predominantly Hispanic population, which may influence external applicability. Given the limited sample size, subgroup analyses comparing syndromic and non-syndromic patients were not performed, and potential differences in pulmonary function related to connective tissue disorders could not be evaluated. Additionally, we lacked cardiopulmonary exercise testing (CPET) and echocardiographic data, which could have provided more comprehensive assessments of physiological impairment. Lastly, spirometry testing was performed at rest and may not fully capture exertional symptoms commonly reported in patients with pectus excavatum. As FEV₁/FVC ratios were not available, ventilatory patterns (restrictive or obstructive) were not formally classified; therefore, the ROC-derived threshold should be interpreted as a marker of reduced FVC rather than confirmed spirometric restriction.

## Conclusions

Our findings suggest that although a higher Haller index may be weakly associated with mildly reduced spirometric function, this relationship is insufficient to justify its use as a standalone metric for surgical decision-making. The ability of the Haller index to predict functional pulmonary compromise remains limited. A multifactorial approach is warranted, incorporating dynamic cardiopulmonary testing, psychological assessment, and radiologic refinements such as the correction index. Clinicians evaluating pediatric patients with pectus excavatum, particularly in similar demographic settings, should consider these findings when counseling families and determining surgical candidacy, emphasizing comprehensive functional assessment over reliance on radiographic indices alone.
